# Ecological governance and population subjective wellbeing: the mediating role of social trust

**DOI:** 10.3389/fpsyg.2026.1843998

**Published:** 2026-08-03

**Authors:** Mengxia Wen, Rongqing Lu, Wenyuan Sun

**Affiliations:** 1 Shandong University, Jinan, Shandong, China; 2 School of Information Technology, Monash University, Subang Jaya, Selangor, Malaysia; 3 Institute of Systems Science, National University of Singapore, Clementi, Singapore, Singapore

**Keywords:** ecological-philosophy-oriented governance, environmental psychology, mediation analysis, population subjective wellbeing, social trust

## Abstract

Although previous research has shown that governance quality is associated with subjective wellbeing, two gaps remain. First, governance is usually treated as an institutional performance construct, with limited attention to whether governance contexts that combine institutional quality and ecological performance are linked to population wellbeing. Second, the psychosocial pathway underlying this relationship remains underexplored, especially from an environmental psychology perspective that treats social and ecological contexts as conditions of lived wellbeing. This study addresses these gaps by examining ecological-philosophy-oriented governance, defined here as a composite macro-level governance orientation integrating governance quality and ecological performance. Using cross-national secondary data from 312 country-wave observations across 86 countries from 2005 to 2022, drawn from the WVS/EVS, WGI, and EPI, we tested whether social trust mediates the association between ecological-philosophy-oriented governance and population subjective wellbeing. The results showed that ecological-philosophy-oriented governance was positively associated with subjective wellbeing (β = 0.287, *p* < 0.001) and social trust (β = 0.341, *p* < 0.001). Social trust was also positively associated with subjective wellbeing when governance was controlled (β = 0.326, *p* < 0.001), while the governance coefficient was reduced (β = 0.176, *p* < 0.01), a pattern consistent with partial mediation. The indirect effect was significant (0.111, 95% CI [0.063, 0.168]), accounting for about 39% of the total effect. These findings suggest that governance functions not only as an institutional arrangement but also as a socio-environmental context relevant to public wellbeing, with social trust serving as an important linking pathway. The study contributes to environmental psychology by showing that governance quality and ecological performance can be interpreted as macro-level features of the human environment that shape collective subjective wellbeing.

## 1 Introduction

Contemporary governance environments are increasingly shaped by the joint influence of ecological pressure, institutional complexity, social differentiation, and public participation (Folke et al., 2005; Ansell and Gash, 2008). Yet despite a large literature on governance quality and, separately, on subjective wellbeing, three gaps limit current understanding. First, although cross-national studies document that good governance is associated with higher life evaluation, the particular governance profile that arises from combining institutional quality with ecological responsibility—a profile anchored in ecological philosophy—is not analytically distinguished from broader notions such as sustainable governance or environmental governance. Second, the psychosocial mechanism through which governance conditions become lived public experience is seldom modeled explicitly; governance is typically connected to wellbeing as a direct macro-level effect rather than as a contextual pathway that operates through the social-relational climate. Third, empirical work rarely integrates survey-based psychosocial data (e.g., WVS/EVS) with governance indicators (e.g., WGI) and environmental performance indicators (EPI) within a single theoretically interpretable framework (Martinangeli et al., 2024; Verhoest et al., 2024). The present study addresses these gaps by asking whether ecological-philosophy-oriented governance is associated with Population Subjective wellbeing, and whether social trust mediates this relationship.

To situate this contribution, it is useful to clarify what we mean by ecological-philosophy-oriented governance and how it differs from related frameworks. Sustainable governance emphasizes the balancing of economic, social, and environmental goals within a long-term policy horizon but does not foreground a philosophical stance on human–nature relations (Cavalheiro et al., 2025). Environmental governance focuses on the institutional arrangements that regulate environmental resources and outcomes. Post-New Public Management (post-NPM) paradigms such as collaborative governance and public value governance move beyond efficiency and market logic toward coordination, participation, and public-centered delivery, but these paradigms are not primarily framed around human–nature harmony. In contrast, ecological-philosophy-oriented governance is an analytical construct that foregrounds the explicit alignment between governance quality and ecological rationality: it conceives governance as simultaneously institutionally capable and ecologically responsible, and treats this alignment as a socio-environmental context that may reshape how people experience fairness, predictability, belongingness, and long-term security (Zhang and Fu, 2023; Dong and Chen, 2025). This definition provides a clearer conceptual anchor than broad normative appeals to ecological civilization and allows the construct to be operationalized and tested at the cross-national level.

This perspective is important because psychological wellbeing does not emerge solely from individual traits or private life conditions. It is also influenced by the broader governance environment in which people live. In practice, people's wellbeing is closely related to whether governance systems can maintain ecological quality, provide inclusive public services, coordinate social interests, and sustain institutional responsiveness over time. In other words, governance quality is not only a matter of institutional effectiveness, but also a condition that may influence how people evaluate their lives, relate to others, and maintain trust in society. From this standpoint, examining the relationship between governance and psychological wellbeing requires moving beyond narrow institutional indicators toward a broader socio-environmental understanding of governance (Karpouzoglou et al., 2016; May, 2022; Newman et al., 2004; Bennett and Satterfield, 2018).

In the present study, Population Subjective wellbeing is examined at the aggregate rather than the clinical level. More specifically, it is conceptualized as a population-level subjective wellbeing construct reflecting how people evaluate and emotionally experience their lives in a given social context. Empirically, it is represented by aggregated life satisfaction and happiness. This operationalization captures evaluative and affective aspects of collective wellbeing, but it should not be interpreted as a direct measure of psychiatric disorder, clinical mental health, or the full range of psychological functioning. Clarifying this boundary is important because the present study focuses on how governance environments relate to collective subjective wellbeing rather than to individual-level diagnosis or pathology.

Against this background, ecological philosophy offers a particularly meaningful lens for understanding the psychological implications of governance. In contrast to governance paradigms centered primarily on short-term administrative efficiency or economic expansion, ecological philosophy emphasizes harmony between human beings and nature, people-centered inclusiveness, collaborative coordination, and long-term sustainability (Lopes and Farias, 2022; Newig et al., 2018). These principles are not only normatively relevant to social governance, but are also psychologically meaningful. Governance systems informed by ecological philosophy are, in principle, more likely to foster a social environment characterized by inclusion, coordination, and long-term orientation. Such an environment may contribute to psychological wellbeing by improving perceived livability, strengthening social connectedness, and reducing uncertainty about collective development.

This argument also connects the present study with adjacent work in environmental psychology, tourism studies, and quality-of-life research. For example, Ramkissoon (2023) develops a cross-disciplinary model in which perceived social impacts, interpersonal trust, place attachment, pro-social and pro-environmental behavior, and overall quality of life are treated as connected processes. Similarly, Karagöz and Ramkissoon (2023) show that subjective wellbeing can operate within broader meaning-making and behavioral pathways rather than as an isolated outcome. These studies are not about national governance directly, but they are important for the present paper because they show how socio-environmental contexts, relational trust, meaning, and wellbeing can be synthesized across environmental psychology and adjacent social science fields.

Recent empirical work has strengthened different parts of the proposed argument. Helliwell et al. (2018) establish empirical linkages between good governance and national wellbeing using large-scale cross-country data. Martinangeli et al. (2024) provide experimental evidence that institutional quality causes generalized trust, reinforcing the view that governance context reshapes social-relational perceptions. Bi et al. (2025) synthesize cross-sectional and longitudinal evidence that trust and subjective wellbeing are robustly linked across the lifespan. Cross-disciplinary environmental psychology and tourism studies further show that social impacts, interpersonal trust, place attachment, prosociality, meaning in life, and quality of life can be understood as connected wellbeing processes (Ramkissoon, 2023; Karagöz and Ramkissoon, 2023; Ramkissoon, 2022). Despite these contributions, two gaps persist. First, most studies treat ecological philosophy mainly as a normative or policy-oriented discourse rather than examining whether ecological-philosophy-oriented governance is associated with population-level psychological outcomes. Second, although social trust is widely recognized as a core condition of effective governance and social coordination, it is rarely examined as a mediating mechanism linking governance context to psychological wellbeing within an ecological-philosophy-oriented framework.

These gaps are important because the influence of governance on psychological wellbeing is unlikely to be purely direct (Helliwell et al., 2018; Martinangeli et al., 2024). A more plausible interpretation is that governance shapes the social-relational climate within which people interpret institutions, other people, and collective life. From a psychological perspective, social trust can be understood in at least three related ways. First, it functions as a social-relational resource, because trusting environments support belongingness, connectedness, and socially embedded security (Holt-Lunstad, 2024; Oyanedel and Paez, 2021). Second, it operates as an uncertainty-reduction mechanism, because trust lowers perceived unpredictability in everyday interaction and collective life (Breakwell, 2020). Third, it serves as a contextual psychosocial pathway through which governance conditions are translated into lived public experience. In this sense, governance may influence wellbeing partly by shaping whether the surrounding social world is experienced as reliable, fair, and cooperative. When governance systems are more inclusive, coordinated, and environmentally responsible, they may strengthen people's confidence in institutions, improve expectations of reciprocity, and foster a greater sense of social reliability. In turn, stronger social trust may support higher psychological wellbeing by reducing perceived insecurity and reinforcing social connectedness (Bi et al., 2025; Zhang, 2020). Therefore, social trust provides a theoretically meaningful bridge between governance context and population wellbeing.

We focus on social trust rather than alternative candidate mediators (such as perceived inequality, social cohesion, or perceived safety) for three reasons. First, social trust is the mediator most directly implied by our theoretical framing: if ecological-philosophy-oriented governance operates by making the surrounding social world feel reliable, fair, and cooperative, then generalized trust is the most proximate population-level indicator of that perception. Second, social trust has the most extensive cross-national measurement coverage in WVS/EVS, which makes it the only candidate mediator for which country-wave measurement is feasible across the 86 countries and full time range of our matched sample. Third, recent evidence indicates that institutional quality exerts causal effects on generalized trust at the individual level and that trust and subjective wellbeing are robustly and reciprocally linked across the lifespan, making trust a theoretically and empirically defensible primary mediator. We acknowledge, however, that perceived fairness, social cohesion, and institutional trust are plausible complementary mediators and return to this point in the limitations and future-research sections.

At the same time, the study of this relationship still faces a methodological challenge. Existing governance research often relies on static or loosely aggregated indicators, making it difficult to represent governance contexts in a way that is both cross-nationally comparable and psychologically meaningful (Kaufmann et al., 2004; Andriantiatsaholiniaina et al., 2004). What is still lacking is a parsimonious analytical framework that can characterize ecological-philosophy-oriented governance as a macro-level socio-environmental context and then examine its association with Population Subjective wellbeing through a psychologically meaningful social mechanism.

To address this issue, the present study investigates the relationship between ecological-philosophy-oriented governance and Population Subjective wellbeing, with particular attention to the mediating role of social trust. Rather than viewing governance only as an institutional performance outcome, this study conceptualizes ecological-philosophy-oriented governance as a parsimonious socio-environmental macro-context jointly represented by governance quality and ecological performance. From a theoretical perspective, ecological-philosophy-oriented governance may be more likely to coincide with a more inclusive, coordinated, and sustainable public environment, which in turn may be associated with stronger social trust and better Population Subjective wellbeing. The present study is designed as a theory-informed cross-national explanatory association analysis. Its purpose is not to establish strong causal identification, but to examine whether the observed cross-national pattern is consistent with a governance-trust-wellbeing framework derived from ecological philosophy and psychosocial theory.

Based on prior governance, trust, and subjective wellbeing research, the present study develops a directional explanatory expectation and examines whether this expectation is supported in cross-national country-wave data. Accordingly, this study is guided by three research questions.

**RQ1:** Is ecological-philosophy-oriented governance positively associated with Population Subjective wellbeing?

**RQ2:** Is ecological-philosophy-oriented governance positively associated with social trust?

**RQ3:** Does social trust mediate the relationship between ecological-philosophy-oriented governance and Population Subjective wellbeing?

Building on the three research questions and the theoretical reasoning developed above, we examine four directional hypotheses:

**H1:** Ecological-philosophy-oriented governance is positively associated with Population Subjective wellbeing.

**H2:** Ecological-philosophy-oriented governance is positively associated with social trust.

**H3:** Social trust is positively associated with Population Subjective wellbeing, controlling for governance context.

**H4:** Social trust partially mediates the association between ecological-philosophy-oriented governance and Population Subjective wellbeing.

Based on these questions, the contributions of this study are threefold. First, this study extends the literature on ecological philosophy and governance by shifting the focus from institutional and normative interpretation to population-level psychological outcomes. In doing so, it repositions ecological-philosophy-oriented governance not only as a governance paradigm, but also as a socio-environmental context relevant to collective subjective wellbeing.

Second, this study introduces social trust as a key explanatory mechanism linking governance context to wellbeing. This helps clarify how ecological-philosophy-oriented governance may influence psychological outcomes through a social-relational resource, an uncertainty-reduction process, and a contextual psychosocial pathway, rather than only through direct institutional performance.

Third, this study adopts a parsimonious multi-source analytical perspective to represent governance context in a psychologically interpretable way. By combining governance quality and ecological performance within a unified macro-context framework, the study provides a more focused basis for examining the psychological relevance of governance environments than broader but conceptually diffuse multidimensional approaches.

## 2 Related work

Research related to the present study lies at the intersection of ecological philosophy, social governance, psychological wellbeing, social trust, and multi-source contextual assessment. Existing studies have generated important insights into sustainable governance, collaborative governance, and systems-oriented social management. However, these strands of literature have rarely been integrated into a unified research framework for explaining how ecological-philosophy-oriented governance may shape Population Subjective wellbeing through socially embedded psychological mechanisms. To clarify the positioning of this study, the related work is reviewed from four closely connected aspects.

### 2.1 Ecological philosophy, governance, and human-centered development

Ecological philosophy provides an important normative foundation for understanding the relationship among human beings, society, and nature (Sarkar, 2014). Unlike development paradigms that prioritize unilateral economic expansion or governance paradigms centered only on administrative control, ecological philosophy emphasizes harmony between human and nature, coordinated development, people-centered inclusiveness, and the long-term co-evolution of social and environmental systems (Floridi, 2019). Existing studies have shown that ecological philosophy is relevant not only to ecological civilization construction, but also to broader questions of institutional design, public governance, and sustainable development (Fang et al., 2020). In this line of research, the central concern is how governance objectives can be aligned with ecological rationality, social welfare, and long-term resilience.

This perspective is particularly relevant to human-centered development because ecological philosophy does not treat governance merely as an instrument for maintaining order or maximizing economic output (Buckton et al., 2023; Kolandai and Harre, 2025). Instead, it emphasizes the quality of human existence within an integrated social and ecological environment. In this sense, ecological-philosophy-oriented governance may provide a social context that is more conducive to inclusion, livability, social coordination, and long-term security. These characteristics are closely related to the broader conditions under which Population Subjective wellbeing is formed and maintained.

However, most existing studies still treat ecological philosophy primarily as a normative discourse, ideological interpretation, or policy-oriented value framework (Hasan, 2021). Although such studies are important for clarifying the conceptual basis of sustainable governance, they usually stop at the level of principle interpretation rather than examining how ecological-philosophy-oriented governance may be associated with human-centered outcomes in everyday social life (Zhang and Fu, 2023). In many cases, ecological philosophy is introduced as a broad evaluative background, but its principles are not sufficiently connected to measurable public experiences, social perceptions, or wellbeing-related consequences (Dong and Chen, 2025). As a result, while ecological philosophy is frequently discussed in relation to sustainable governance, its potential psychological relevance remains underdeveloped.

Accordingly, the main limitation of this strand of literature is not the lack of value insight, but the limited connection between ecological-philosophy-oriented governance and population-level human outcomes. The present study builds on the normative basis of ecological philosophy while extending the discussion toward psychological wellbeing. The literature establishes the normative relevance of ecological philosophy for governance but does not test whether an ecologically and people-centered governance profile is systematically linked to population-level subjective wellbeing. The literature establishes the normative relevance of ecological philosophy for governance, but it still stops largely at conceptual or policy-oriented discussion. What remains insufficiently tested is whether an ecologically and people-centered governance profile is systematically associated with population-level subjective wellbeing. This gap directly motivates

**H1**: ecological-philosophy-oriented governance is positively associated with Population Subjective wellbeing.

### 2.2 Governance context and population subjective wellbeing

Social governance has been widely studied as a process involving state institutions, market actors, social organizations, and citizens. Compared with traditional government-centered management, contemporary governance research increasingly emphasizes co-construction, collaborative participation, distributed responsibility, and adaptive coordination (Wang and Ran, 2023). Existing studies have examined grassroots governance, digital governance, public participation, governance modernization, and social risk management (Allen et al., 2023). Related research has shown that governance outcomes depend not only on top-down policy implementation, but also on the interaction among institutional authority, social autonomy, and public engagement (Raschendorfer and Roder Figueira, 2024).

These studies provide an important basis for understanding governance as a social environment rather than merely an administrative arrangement (Asthana et al., 2024; Fabian et al., 2023). From a psychological perspective, the governance environment matters because it influences whether people experience daily life as secure, fair, orderly, inclusive, and socially coordinated. Governance systems that provide effective public services, maintain ecological quality, reduce uncertainty, and support collective participation may contribute to more favorable conditions for Population Subjective wellbeing. In this sense, governance context is not only an institutional variable, but also a background condition that may shape how people perceive their lives and social world.

This interpretation requires synthesis across disciplines. In environmental psychology and tourism research, quality of life is often explained through the perceived social impacts of place-based development, trust, attachment, pro-social behavior, and pro-environmental behavior rather than through objective conditions alone (Ramkissoon, 2023). Research on nostalgic emotions similarly shows that subjective wellbeing is embedded in meaning-related and behavior-related psychological pathways (Karagöz and Ramkissoon, 2023). These findings suggest that macro-contexts matter psychologically when they are translated into social meanings, relational expectations, and perceived life quality. The present study extends this logic from community and tourism settings to the cross-national governance context.

Recent cross-national work confirms the empirical plausibility of this proposition. Karataş (2026) shows, through a cross-indicator analysis of OECD countries, that governance quality is a robust determinant of quality of life and is positively linked to life satisfaction and several other welfare dimensions. Helliwell et al. (2018) similarly document strong empirical linkages between good governance and national wellbeing across survey waves, while Fabian et al. (2023) argue that wellbeing-oriented public policy should respect the subjective experience of the governed rather than rely only on a planner-centric perspective. Taken together, these studies support the view that governance context functions as a psychologically relevant background condition rather than merely an institutional variable.

Nevertheless, most current studies still focus on institutional arrangements, case-based interpretation, governance innovation, or performance-oriented evaluation (Manoharan et al., 2023). Although these studies clarify how governance operates, they rarely investigate governance as a context associated with psychological wellbeing. In particular, existing research seldom examines whether ecologically informed and people-centered governance environments are linked to better population-level psychological outcomes (Antonini and Achilli, 2025; Asthana et al., 2024). This limitation becomes more evident when governance is examined from a systems perspective, because the key issue is not only whether institutions function effectively, but also whether they create a social environment supportive of human wellbeing.

Accordingly, the gap in this stream of literature lies in the insufficient linkage between governance research and psychological outcome research. The present study addresses this issue by conceptualizing ecological-philosophy-oriented governance as a socio-environmental context relevant to Population Subjective wellbeing. The literature shows that governance context matters for wellbeing but has not specifically examined whether ecologically and people-centered governance profiles are associated with a more trusting social climate. Prior research suggests that governance context matters for quality of life and subjective wellbeing, yet it has rarely isolated the specific governance profile examined here, namely the joint alignment of governance quality and ecological responsibility. The remaining gap is therefore not whether governance matters in general, but whether ecological-philosophy-oriented governance is associated with a more favorable psychosocial climate. This gap directly motivates

**H2**: ecological-philosophy-oriented governance is positively associated with social trust.

### 2.3 Social trust as a relational mechanism linking governance and wellbeing

With the increasing complexity of governance environments, a growing body of research has called for systems-oriented approaches to public governance, social management, and sustainable development (Smith et al., 2005). From this perspective, governance should be understood as a complex socio-technical or socio-environmental system characterized by nonlinearity, interdependence, feedback, and multi-level interaction (Muñoz-Erickson et al., 2016). Systems-oriented governance studies have emphasized that governance outcomes emerge from the coupled effects of institutional structure, actor behavior, resource allocation, environmental constraints, and social response (Krueger et al., 2022). These studies are valuable because they shift attention from isolated institutional inputs to broader relational and contextual conditions. This perspective is especially useful for understanding the psychological role of social trust. From a psychological standpoint, trust is not merely a background feature of governance systems, but a socially embedded resource that helps individuals navigate collective life. First, social trust can be understood as a social-relational resource, because trusting environments support belongingness, connectedness, and a sense of socially embedded security. Second, trust functions as an uncertainty-reduction mechanism, because it lowers perceived unpredictability in everyday interaction and collective life. Third, trust serves as a contextual psychosocial pathway through which macro-level governance conditions may be translated into lived public experience. This interpretation is consistent with research showing that social bonds and prosocial behavior can support resilience, mental wellbeing, overall wellbeing, and quality of life in periods of separation and loss (Ramkissoon, 2022). It also aligns with place-based quality-of-life models in which interpersonal trust helps connect perceived social impacts with attachment, pro-social behavior, pro-environmental behavior, and support for sustainable development (Ramkissoon, 2023). In this sense, governance may matter for wellbeing partly because it shapes whether the surrounding social world is experienced as reliable, fair, and cooperative.

Recent empirical research supports the bridging role of social trust. Martinangeli et al. (2024) show experimentally that institutional quality causes generalized trust, providing causal evidence for the governance → trust pathway. Bi et al. (2025) meta-analyze cross-sectional and longitudinal evidence and report robust associations between trust and subjective wellbeing across age groups, supporting the trust → wellbeing pathway. Verhoest et al. (2024) further demonstrate that trust and distrust play distinct roles in regulatory governance relations. The same relational logic is visible in environmental psychology and tourism-related work, where trust and pro-social behavior are treated as mechanisms through which social conditions become quality-of-life outcomes (Ramkissoon, 2023, 2022).

In governance contexts characterized by stronger coordination, better responsiveness, greater inclusion, and more consistent ecological responsibility, people may be more likely to develop generalized expectations that institutions and other people are dependable and worthy of confidence (Wei et al., 2022; Verhoest et al., 2024). In turn, this kind of social trust may support psychological wellbeing by reducing insecurity, strengthening social connectedness, and improving the perceived stability of collective life. The relevance of trust therefore lies not only in its institutional meaning, but also in its psychosocial role in structuring how people experience everyday social environments. Although existing governance studies have highlighted collaborative structures, public participation, and cross-actor coordination (Tukker and Butter, 2007), they have rarely incorporated social trust as an explicit explanatory mechanism connecting governance context to population wellbeing. Many studies remain at the macro-theoretical level, while others focus on structural governance components without considering how these components may be psychologically translated into public experience (Sol et al., 2018; Geels et al., 2023). As a result, current literature is limited in its ability to explain not only whether governance matters, but also through what psychosocial mechanism governance may influence psychological wellbeing.

Therefore, the limitation of this line of research is not the lack of systems thinking, but the insufficient integration of psychologically meaningful relational mechanisms. The present study addresses this issue by introducing social trust as a mediating pathway between ecological-philosophy-oriented governance and Population Subjective wellbeing. While the two halves of the causal chain—governance → trust and trust → wellbeing—have each received empirical support, they have rarely been integrated within a unified mediation framework for ecological-philosophy-oriented governance. The two halves of the proposed pathway have each received empirical support in prior work: governance conditions shape generalized trust, and trust is positively associated with subjective wellbeing. However, these two strands have rarely been integrated into a unified mediation framework centered on ecological-philosophy-oriented governance. This gap directly motivates

**H3**: social trust is positively associated with Population Subjective wellbeing, and

**H4**: social trust partially mediates the association between ecological-philosophy-oriented governance and Population Subjective wellbeing.

### 2.4 Multi-source representation of governance context

Research on governance has increasingly emphasized the need to move beyond single-source or single-dimensional indicators when characterizing social environments. Governance contexts are not defined only by institutional performance in a narrow administrative sense (Cavalheiro et al., 2025; Aggarwal and Anderies, 2023). They are also shaped by broader ecological and public-life conditions that influence how collective environments are organized and experienced. For this reason, a multi-source representation is often more suitable than a single institutional metric when the goal is to examine whether governance context is related to human-centered outcomes such as social trust or Population Subjective wellbeing. At the same time, not all multi-source governance representations are equally appropriate for psychologically oriented research. Existing quantitative studies have often focused on governance scoring, structural classification, or methodological sophistication, including fuzzy evaluation and other data-driven assessment tools (Papageorgiou and Salmeron, 2012; Klassen et al., 2023; Targetti et al., 2021). Although these approaches are useful for describing complex governance systems, they are typically designed for governance-centered evaluation rather than for explaining psychologically meaningful public outcomes. As a result, the link between governance representation and human experience often remains underdeveloped.

For the purposes of the present study, the key issue is therefore not how to construct the most technically elaborate governance index, but how to represent governance context in a way that is theoretically focused, cross-nationally comparable, and psychologically interpretable (Tang et al., 2025; Balázsi et al., 2019). In particular, if governance is examined as a socio-environmental context relevant to population subjective wellbeing, then its representation should capture not only governance quality in the institutional sense, but also ecological conditions that shape everyday collective life. This is the rationale for adopting a parsimonious multi-source representation of ecological-philosophy-oriented governance in the present study. Rather than relying on a broad and conceptually diffuse multidimensional structure, the study combines governance quality and ecological performance to characterize a macro-level context that is relevant to social trust and Population Subjective wellbeing. In this sense, the value of multi-source representation here lies not in methodological complexity itself, but in providing a more psychologically meaningful basis for examining how governance environments may be associated with collective wellbeing. A parsimonious WGI+EPI representation is theoretically appropriate for examining the psychological relevance of governance environments and is more interpretable than broader multidimensional indices when the outcome of interest is population-level subjective wellbeing. The methodological issue is not simply how to build a more complex governance index, but how to construct a governance representation that is theoretically focused, cross-nationally comparable, and psychologically interpretable. This justifies the use of a parsimonious WGI+EPI representation in the present study and clarifies why the construct is designed as a macro-context proxy rather than an exhaustive governance score.

### 2.5 Summary of research gap and positioning of this study

Overall, the existing literature has established important foundations for understanding ecological philosophy, collaborative governance, systems-oriented social management, and data-driven contextual assessment. However, a clear gap remains at their intersection. Current studies rarely provide an integrated framework that simultaneously: (1) treats ecological-philosophy-oriented governance as a measurable socio-environmental context rather than only a normative discourse; (2) examines Population Subjective wellbeing as an important human-centered outcome of governance; and (3) explains the governance–wellbeing relationship through a socially embedded relational mechanism such as social trust.

This gap is precisely where the present study is positioned. Different from prior work, this study moves beyond treating ecological philosophy as a purely conceptual background and instead considers ecological-philosophy-oriented governance as a contextual condition relevant to population wellbeing. It also moves beyond governance performance analysis by introducing psychological wellbeing as a substantive outcome variable. Finally, it moves beyond direct governance–outcome association by examining the mediating role of social trust.

In this sense, the contribution of the present study is not merely to extend governance evaluation toward another application setting. Rather, it re-frames ecological-philosophy-oriented governance as a psychologically meaningful socio-environmental context and provides a more integrated explanation of how governance may be associated with Population Subjective wellbeing through social trust.

## 3 Methodology

To test the proposed hypotheses, this study develops a multi-source analytical framework that represents ecological-philosophy-oriented governance as a multidimensional socio-environmental context and examines its association with Population Subjective wellbeing through the mediating role of social trust. Different from conventional governance studies that focus primarily on institutional scoring or static policy evaluation, the present study treats governance as a contextual condition that may shape the lived social environment of the population. Accordingly, the methodological design combines multi-source governance representation, population-level trust and wellbeing measurement, and mediation-oriented statistical analysis within a unified framework.

### 3.1 Analytical design overview

The analytical design contains three tightly connected stages. First, ecological-philosophy-oriented governance is represented as a parsimonious macro-level socio-environmental context based on two public indicator systems: governance quality and ecological performance. Specifically, governance quality is captured using the Worldwide Governance Indicators, and ecological performance is captured using the Environmental Performance Index. Second, social trust and Population Subjective wellbeing are measured as population-level social-psychological constructs derived from public survey data. Third, the empirical analysis tests both the direct relationship between governance context and psychological wellbeing and the indirect pathway operating through social trust. Following the theoretical framework, ecological-philosophy-oriented governance is treated as the explanatory variable, social trust as the mediator, and Population Subjective wellbeing as the focal outcome. This design allows the study to move beyond purely descriptive governance assessment and instead examine whether a governance context grounded in ecological and people-centered principles is associated with psychologically meaningful collective outcomes.

### 3.2 Representation of ecological-philosophy-oriented governance

The first component of the methodology is the representation of ecological-philosophy-oriented governance. In the present study, this construct is represented through a parsimonious combination of governance quality and ecological performance. This operationalization reflects the premise that ecological-philosophy-oriented governance should capture both the institutional capacity of public governance and the ecological orientation of social development. Rather than treating governance as a broad and conceptually diffuse multidimensional structure, the present study focuses on a more interpretable macro-context design that is suitable for psychological-outcome analysis.

Let the governance-context score for country *c* in year *t* be denoted by *G*_*c,t*_. The index is constructed from two components: a governance component derived from WGI and an ecological component derived from EPI. All indicators are first direction-aligned so that larger values consistently represent more favorable governance or ecological conditions. They are then normalized to ensure comparability across sources.

The governance component is defined as


$$\begin{array}{l} GOV_{c,t}=\frac{1}{J}\overset{J}{\underset{j=1}{\sum }}{\tilde{g}}_{c,t}^{\left(j\right)}, \end{array}$$

where ${\tilde{g}}_{c,t}^{\left(j\right)}$ denotes the normalized score of governance indicator *j* for country *c* in year *t*, and *J* is the number of retained WGI dimensions.

The ecological component is defined as


$$\begin{array}{l} ECO_{c,t}=\frac{1}{Q}\overset{Q}{\underset{q=1}{\sum }}{\tilde{e}}_{c,t}^{\left(q\right)}, \end{array}$$

where ${\tilde{e}}_{c,t}^{\left(q\right)}$ denotes the normalized score of ecological indicator *q* for country *c* in year *t*, and *Q* is the number of retained EPI dimensions.

The overall ecological-philosophy-oriented governance index is then defined as


$$\begin{array}{l} G_{c,t}=\frac{1}{2}\left(GOV_{c,t}+ECO_{c,t}\right). \end{array}$$

Higher values of *G*_*c,t*_ indicate that the country-year context is characterized by stronger governance quality and stronger ecological performance, and is therefore more consistent with the logic of ecological-philosophy-oriented governance.

In the main specification, the governance component prioritizes WGI dimensions most closely related to coordinated and people-centered governance, namely Government Effectiveness, Rule of Law, and Voice and Accountability. The ecological component prioritizes broad EPI dimensions that reflect environmental quality and sustainability conditions, namely Environmental Health, Ecosystem Vitality, and Climate Change Performance. This representation is intentionally parsimonious. Its purpose is not to exhaust all possible dimensions of governance, but to provide a theoretically focused and psychologically interpretable macro-context measure for examining the governance–trust–wellbeing relationship. This measure should therefore be interpreted as a theoretically focused macro-context proxy rather than a full representation of all dimensions of ecological-philosophy-oriented governance.

The equal-weight scheme is adopted for three reasons. Theoretically, ecological-philosophy-oriented governance is conceptualized as the joint realization of institutional capacity and ecological orientation, with neither dimension prior to the other; assigning pre-specified unequal weights would impose an implicit hierarchy that is not warranted by the theory. Empirically, equal weighting is a transparent and replicable benchmark widely used in composite social-environmental indices when the analyst lacks strong external evidence about the correct weights (Centre, 2008); it is less sensitive to overfitting than data-driven weighting when the final model is also estimated on the same sample. Psychometrically, we performed an exploratory factor analysis on the six direction-aligned, normalized component indicators across the 312 country-wave observations. Results show that the first principal factor explains about 68% of the common variance, and all six components load substantially on it (loadings > 0.55), which is consistent with the interpretation that WGI and EPI components capture facets of a single latent macro-context. A composite that equally weights the two conceptual blocks (WGI and EPI) is therefore a defensible parsimonious proxy. To guard against model dependence, supplementary specifications in re-estimate the index using 0.6/0.4 and 0.4/0.6 weightings between WGI and EPI, and perform a leave-one-subcomponent-out sensitivity analysis. Results are substantively unchanged.

### 3.3 Measurement of social trust

Social trust is treated as the mediating variable linking governance context to Population Subjective wellbeing. In the present framework, social trust is conceptualized as a population-level psychosocial resource reflecting generalized expectations that other people and the broader social environment are reliable, fair, cooperative, and worthy of confidence. This conceptualization is psychologically relevant because it captures the degree to which everyday collective life is experienced as socially predictable and relationally secure. In this sense, social trust is not treated merely as a governance-related attitude, but as a contextual psychosocial pathway through which macro-level governance conditions may be translated into population-level wellbeing outcomes.

Because the study focuses on population-level outcomes, social trust is operationalized at the country-wave level. Let *T*_*c,t*_ denote the social-trust level of country *c* in survey wave *t*. When a multi-item trust representation is available, the trust index is defined as


$$\begin{array}{l} T_{c,t}=\frac{1}{K}\overset{K}{\underset{k=1}{\sum }}{\tilde{t}}_{c,t}^{\left(k\right)}, \end{array}$$

where ${\tilde{t}}_{c,t}^{\left(k\right)}$ denotes the normalized score of trust-related item *k*, and *K* is the total number of retained trust indicators. Larger values indicate a higher level of social trust.

This operationalization should therefore be interpreted as a population-level indicator of generalized social trust. It captures a psychosocial climate of perceived reliability and social predictability rather than a clinical or personality-based trust disposition at the individual level.

### 3.4 Measurement of population subjective wellbeing

Population Subjective wellbeing is the dependent variable of the study. In the present framework, it is conceptualized as a population-level subjective wellbeing construct that reflects how people evaluate and emotionally experience their lives within a given social context. This definition is intentionally narrower than broad notions of psychological health or mental functioning. More specifically, the construct is designed to capture aggregate evaluative and affective aspects of collective wellbeing rather than clinical mental health, psychiatric disorder, or the full range of psychological functioning. Because the present study is conducted at the population level, the outcome variable is measured through aggregate survey-based indicators rather than individual diagnosis or symptom-based assessment.

Let *W*_*c,t*_ denote the Population Subjective wellbeing level of country *c* in survey wave *t*. In the present study, this variable is represented through publicly available wellbeing indicators that reflect population-level life evaluation and affective appraisal. The empirical implementation prioritizes life satisfaction and happiness because these two indicators provide a parsimonious and conceptually coherent representation of subjective wellbeing at the aggregate level. When more than one wellbeing indicator is available, a normalized composite index is constructed. When only one aggregate indicator is available, it is directly used as the outcome after normalization.

When a multi-item representation is available, the wellbeing index is defined as


$$\begin{array}{l} W_{c,t}=\frac{1}{M}\overset{M}{\underset{m=1}{\sum }}{\tilde{w}}_{c,t}^{\left(m\right)}, \end{array}$$

where ${\tilde{w}}_{c,t}^{\left(m\right)}$ is the normalized value of wellbeing-related item *m*, and *M* is the total number of retained subjective wellbeing indicators. Larger values indicate higher levels of Population Subjective wellbeing.

This operationalization should therefore be interpreted as a parsimonious population-level proxy for collective subjective wellbeing. It is appropriate for cross-national comparative analysis of governance context, but it should not be interpreted as a direct measure of clinical mental health or individual psychiatric status.

### 3.5 Empirical strategy

The empirical strategy proceeds in three steps using country-wave observations.

First, the direct association between ecological-philosophy-oriented governance and Population Subjective wellbeing is estimated:


$$\begin{array}{l} W_{c,t}=\alpha_{0}+\alpha_{1}G_{c,t}+\alpha_{2}^{⊤}{\text{C}}_{c,t}+\varepsilon_{c,t}. \end{array}$$

A significantly positive α_1_ supports Hypothesis 1.

Second, the relationship between ecological-philosophy-oriented governance and social trust is estimated:


$$\begin{array}{l} T_{c,t}=\beta_{0}+\beta_{1}G_{c,t}+\beta_{2}^{⊤}{\text{C}}_{c,t}+\mu_{c,t}. \end{array}$$

A significantly positive β_1_ supports Hypothesis 2.

Third, the mediation model is estimated by jointly including governance context and social trust in the wellbeing equation:


$$\begin{array}{l} W_{c,t}=\gamma_{0}+\gamma_{1}G_{c,t}+\gamma_{2}T_{c,t}+\gamma_{3}^{⊤}{\text{C}}_{c,t}+\xi_{c,t}. \end{array}$$

A significantly positive γ_2_ supports Hypothesis 3. If β_1_ and γ_2_ are both significant, and the coefficient of *G*_*c,t*_ decreases in magnitude after the inclusion of *T*_*c,t*_, this provides evidence for the mediating role of social trust, thereby supporting Hypothesis 4.

To formally test the indirect effect, the product term β_1_ × γ_2_ is evaluated using bootstrap confidence intervals. Mediation is considered statistically supported when the bootstrapped indirect effect is significantly different from zero. In the present study, mediation analysis is used as an explanatory modeling strategy for evaluating whether the observed cross-national pattern is consistent with the proposed psychosocial pathway. Throughout the manuscript, mediation analysis is used as an *explanatory modeling strategy* for evaluating whether the observed cross-national pattern is consistent with the proposed psychosocial pathway. The estimates should be read as cross-national associations consistent with a governance–trust–wellbeing framework rather than as identified causal effects.

### 3.6 Causal limitations, endogeneity, and the choice of estimator

Because the estimator is a cross-national mediation model on country-wave data, we explicitly discuss three potential sources of bias. Among the 312 analytical observations, 241 (≈77%) are aligned to the exact survey year, while 71 rely on the nearest adjacent year when exact-year overlap between WVS/EVS waves and macro indicators is unavailable. Because WGI and EPI indicators are slow-moving at the country level, such one- or two-year gaps are unlikely to generate large attenuation bias, but they may still introduce classical measurement error in *G*_*c,t*_. A dedicated robustness check (Section 5.5) re-estimates all models on the exact-year-matched subsample and on a ±1-year window; results are substantively unchanged.

Reverse causality cannot be ruled out: societies with higher aggregate wellbeing and stronger generalized trust may also develop better governance and environmental performance over time. We therefore frame the estimates as cross-national associations consistent with a governance–trust–wellbeing framework rather than as identified causal effects. Nonetheless, three design features reduce the risk that the observed pattern is purely an artifact of reverse causation. First, *G*_*c,t*_ is measured with ecological and governance indicators that are largely shaped by long-run institutional and environmental processes and are less responsive to short-run fluctuations in aggregate wellbeing. Second, we control for GDP per capita (log), urbanization, and age structure, which absorb part of the confounding variation with long-run development.

Because many countries appear only once or twice in the matched WVS/EVS waves retained in our window (the dataset is highly unbalanced and country-wave rather than strict panel), country fixed effects would absorb most of the between-country variation that is theoretically of interest. WGI and EPI vary slowly within countries and would leave only a small amount of short-run variation to identify α_1_ and β_1_. We therefore treat the pooled cross-national specification as the main model and conduct two complementary checks. First, we cluster standard errors at the country level to account for within-country dependence. Second, we estimate a random-effects (Mundlak-style) specification as a supplementary check; signs and significance of the main coefficients are preserved, though magnitudes shrink slightly, consistent with the view that a nontrivial share of the variation is between countries. This design choice is therefore substantively motivated: the theoretical claim concerns differences across governance environments, not within-country fluctuations.

We note two further analytical choices that relate to the design. First, regarding cross-country heterogeneity: country-level aggregation may conceal within-country differences across regions, income groups, or cultural subgroups. The present design is therefore appropriate for characterizing between-country variation in governance context but should not be read as describing within-country inequalities, a point we revisit in the Limitations section. Second, regarding covariate selection: we prioritize controls that are theoretically implicated in both governance quality and subjective wellbeing (economic development, urbanization, age structure), avoid controls that are likely to be mechanisms rather than confounders (e.g., institutional trust, which we treat as a related but distinct construct from generalized trust), and report a supplementary analysis (Section 4.7) that includes education, inequality, and institutional trust in a reduced subsample.

### 3.7 Robustness and implementation details

Several robustness checks are conducted to evaluate the stability of the findings. First, alternative formulations of the ecological-philosophy-oriented governance index are estimated, including different weighting schemes for the WGI and EPI components. In addition to the baseline equal-weight specification, supplementary versions assigning relatively greater weight to governance quality or ecological performance are considered. Second, alternative wellbeing constructions are examined by reconstructing the dependent variable using life satisfaction only and happiness only, respectively. Third, the mediation analysis is re-estimated after excluding observations with relatively large year-matching gaps between survey waves and macro-level indicators.

Most importantly, a leave-one-subcomponent-out sensitivity analysis is conducted to assess whether the governance index is disproportionately driven by any single retained WGI or EPI component. In this procedure, the ecological-philosophy-oriented governance index is sequentially reconstructed after omitting one component at a time from the baseline set of retained dimensions, namely Government Effectiveness, Rule of Law, Voice and Accountability, Environmental Health, Ecosystem Vitality, and Climate Change Performance. The main regression and mediation models are then re-estimated under each reduced specification. This procedure allows the study to assess whether the observed governance–trust–wellbeing relationship is robust to plausible changes in index composition and is not driven by a single retained subcomponent.

All indicators are direction-aligned and normalized before index construction and model estimation. Missing values are handled through a transparent preprocessing procedure. When short gaps are limited and adjacent observations are available, interpolation may be used; otherwise, severely incomplete observations are excluded. The empirical analysis is implemented using standard statistical software. Composite indices are constructed after preprocessing, and the mediation models are estimated using ordinary least squares or panel-based regression specifications depending on the final data structure. The mediation models are estimated using ordinary least squares with country-clustered standard errors. The indirect effect β_1_ × γ_2_ is evaluated using nonparametric bootstrap with 5,000 resamples and bias-corrected confidence intervals. All reported coefficients are accompanied by standard errors and significance tests, and all focal variables are standardized so that coefficients may be read as standardized effect sizes. Mediation is considered empirically supported when the 95% bootstrap confidence interval of the indirect effect does not include zero; this criterion is applied consistently in the main analysis and in all robustness specifications.

## 4 Data sources and variable construction

### 4.1 Data sources

To empirically test the proposed framework, this study adopts a parsimonious public-data design that combines cross-national survey data with macro-level governance and environmental indicators. The overall data structure follows a context–mechanism–outcome logic. Specifically, ecological-philosophy-oriented governance is represented through national governance quality and environmental performance, social trust is measured using public survey-based trust items, and Population Subjective wellbeing is measured using public survey-based wellbeing indicators.

For the trust and psychological wellbeing side, this study uses the integrated World Values Survey/European Values Study (WVS/EVS) public data. WVS/EVS provides cross-national multi-wave survey data and includes long-running items on generalized trust, happiness, and life satisfaction, which makes it particularly suitable for constructing population-level measures of social trust and psychological wellbeing (Luijkx et al., 2022).

For the governance-context side, this study uses the Worldwide Governance Indicators (WGI) published by the World Bank. WGI provides internationally comparable annual indicators for multiple dimensions of governance quality, including government effectiveness, rule of law, and voice and accountability, which are especially relevant to the present study's people-centered and coordination-oriented understanding of governance (Kaufmann et al., 2004).

To capture the ecological dimension of ecological-philosophy-oriented governance, this study uses the Environmental Performance Index (EPI). EPI provides cross-national environmental performance indicators and is designed to support comparative evaluation of environmental quality and sustainability-related conditions (Wolf et al., 2022). The public datasets used in this study and their roles in the analytical framework are summarized in Table 1.

**Table 1:** Summary of data sources used in the study.

Data source	Main role in this study	Key variables/dimensions
WVS/EVS	Social-psychological survey source	Social trust, happiness, life satisfaction
WGI	Governance-context source	Government effectiveness, rule of law, voice and accountability
EPI	Ecological-context source	Environmental health, ecosystem vitality, climate-related performance

Because the present study relies exclusively on publicly available and anonymized secondary data, no new participant recruitment, intervention, or personally identifiable information is involved.

### 4.2 Dataset construction

The preferred analytical unit is the country-wave observation. For the trust and psychological wellbeing side, the empirical analysis uses the integrated WVS/EVS public data and retains country-wave observations from the survey waves that provide valid information on generalized trust, life satisfaction, and happiness. In the present matched sample, the retained country-wave observations span the period from 2005 to 2022, depending on country-specific survey availability. This design allows the study to combine cross-national survey-based psychosocial information with macro-level governance and ecological indicators while preserving a country-wave analytical structure.

For each country participating in a retained WVS/EVS survey wave, individual-level responses are aggregated to the country-wave level and then matched to the corresponding macro-level governance and ecological indicators. The final matched sample is constructed in five steps. First, the pooled WVS/EVS data are screened to retain only country-wave observations with valid responses on generalized trust, life satisfaction, and happiness. This step yields 356 country-wave observations. Second, individual-level responses are aggregated to the country-wave level to construct population-level trust and wellbeing indicators. Third, the aggregated country-wave observations are matched to the corresponding WGI governance indicators. Among these observations, 248 are matched using the exact survey year and 76 are matched using the nearest adjacent year, resulting in 324 observations with valid governance information. Fourth, the matched observations are linked to the corresponding EPI ecological indicators. After this step, 318 country-wave observations remain with non-missing governance and ecological context information. Fifth, observations with severe missingness in the focal variables or control variables are excluded, yielding the final analytical sample of 312 country-wave observations from 86 countries.

This procedure ensures that each analytical observation contains a governance-context measure, a social-trust measure, and a Population Subjective wellbeing measure. Among the final 312 observations, 241 are aligned to the exact survey year, whereas 71 rely on the nearest adjacent year when exact-year overlap between survey and macro-level indicators is unavailable. The overall dataset construction procedure is further summarized in Table 2.

**Table 2:** Dataset construction workflow, matching procedure, and sample retention.

Step	Description	Remaining observations
Step 1	Extract country-wave observations from the integrated WVS/EVS data with valid responses on generalized trust, life satisfaction, and happiness.	356
Step 2	Aggregate individual-level survey responses to the country-wave level.	356
Step 3	Match each country-wave observation with the corresponding WGI governance indicators for the same survey year or, when unavailable, the nearest adjacent year.	324
Step 4	Match each country-wave observation with the corresponding EPI ecological indicators for the same survey year or, when unavailable, the nearest adjacent year.	318
Step 5	Remove observations with severe missingness after matching and retain the final analytical sample for mediation analysis.	312

Nearest-year matching can introduce classical measurement error in *G*_*c,t*_ and attenuate the estimated association with trust and wellbeing. Three features of the design limit this concern. First, WGI and EPI indicators are slow-moving at the country level and small year-differences translate into small changes in the composite score. Second, 77% of observations are exact-year matches and the analysis is reproduced on this subsample without material change in conclusions. Third, a bounds-style check that drops all observations with matching gaps greater than one year yields qualitatively identical results. These patterns are consistent with attenuation (rather than bias of a specific sign) being small in this dataset.

### 4.3 Independent variable: ecological-philosophy-oriented governance

The independent variable is ecological-philosophy-oriented governance. In the present study, this construct is represented through a parsimonious combination of governance quality and ecological performance. The conceptual rationale is that ecological-philosophy-oriented governance should reflect both the institutional capacity of public governance and the ecological orientation of national development.

Let *G*_*c,t*_ denote the ecological-philosophy-oriented governance score of country *c* in year *t*. The index is constructed from two components: a governance component derived from WGI and an ecological component derived from EPI. The governance component is defined as the average of selected WGI dimensions:


$$\begin{array}{l} GOV_{c,t}=\frac{1}{J}\overset{J}{\underset{j=1}{\sum }}{\tilde{g}}_{c,t}^{\left(j\right)}, \end{array}$$

where ${\tilde{g}}_{c,t}^{\left(j\right)}$ denotes the normalized score of WGI governance dimension *j*, and *J* is the number of WGI dimensions retained in the main specification. The ecological component is defined as:


$$\begin{array}{l} ECO_{c,t}=\frac{1}{Q}\overset{Q}{\underset{q=1}{\sum }}{\tilde{e}}_{c,t}^{\left(q\right)}. \end{array}$$

where ${\tilde{e}}_{c,t}^{\left(q\right)}$ denotes the normalized ecological performance indicator *q* derived from EPI, and *Q* is the number of ecological indicators retained. The final ecological-philosophy-oriented governance index is then defined as


$$\begin{array}{l} G_{c,t}=\frac{1}{2}\left(GOV_{c,t}+ECO_{c,t}\right). \end{array}$$

Higher values of *G*_*c,t*_ indicate that the country-year context is characterized by stronger governance quality and stronger ecological performance, and is therefore more consistent with the logic of ecological-philosophy-oriented governance. In the main specification, the WGI component prioritizes dimensions most closely related to coordinated and people-centered governance, namely Government Effectiveness, Rule of Law, and Voice and Accountability. The EPI component prioritizes broad ecological performance indicators that reflect environmental quality and sustainability conditions, namely Environmental Health, Ecosystem Vitality, and Climate Change Performance. All component indicators are direction-aligned and normalized before aggregation.

### 4.4 Mediator: social trust

The mediator is social trust. Generalized trust is prioritized because it provides the most comparable cross-national indicator of broad social reliability, although it does not exhaust other forms of trust such as institutional trust or domain-specific interpersonal trust. In the preferred specification, social trust is measured using the generalized-trust item from WVS/EVS. This item captures whether respondents believe that most people can be trusted and is widely used as a cross-national indicator of generalized social trust. Let *T*_*c,t*_ denote the social-trust level of country *c* in survey wave *t*. The country-wave trust score is constructed as the mean of the normalized individual trust responses within that country-wave:


$$\begin{array}{l} T_{c,t}=\frac{1}{N_{c,t}}\overset{N_{c,t}}{\underset{i=1}{\sum }}{\tilde{t}}_{i,c,t}, \end{array}$$

where ${\tilde{t}}_{i,c,t}$ is the normalized trust response of individual *i* in country *c* and wave *t*, and *N*_*c,t*_ is the number of valid respondents in that country-wave. Higher values of *T*_*c,t*_ indicate stronger generalized social trust.

Generalized trust is prioritized because it offers the broadest cross-national coverage across WVS/EVS waves. We acknowledge three limitations. First, a single-item measure cannot capture multiple facets of trust simultaneously (e.g., institutional trust, interpersonal trust toward specific groups, political trust). Second, the dichotomous framing of the generalized trust item may invite cross-cultural response differences. Third, when a multi-item representation is available we aggregate it via the averaging equation, but where only the single item is available the construct is inevitably narrower. To partially address these concerns, we discuss reverse causality and confounding explicitly and recommend in the conclusion that future work triangulate with institutional trust and domain-specific trust indicators.

### 4.5 Dependent variable: population subjective wellbeing

The dependent variable is Population Subjective wellbeing. In the main specification, this variable is operationalized as a population-level subjective wellbeing index constructed from WVS/EVS items on life satisfaction and happiness. These two indicators capture complementary aspects of subjective wellbeing: life satisfaction reflects the evaluative appraisal of one's life, whereas happiness reflects a more affective appraisal. Their aggregation therefore provides a parsimonious representation of collective subjective wellbeing at the country-wave level. This operationalization is intentionally narrower than broad notions of psychological health. It is designed to capture evaluative and affective aspects of population wellbeing rather than clinical mental health, psychiatric disorder, or the full range of psychological functioning. Accordingly, the dependent variable in this study should be interpreted as a population-level proxy for collective subjective wellbeing rather than as a direct indicator of individual pathology or diagnosis.

Let *W*_*c,t*_ denote the Population Subjective wellbeing level of country *c* in survey wave *t*. When both life satisfaction and happiness are available, the country-wave wellbeing index is defined as


$$\begin{array}{l} W_{c,t}=\frac{1}{2}\left({\tilde{l}s}_{c,t}+{\tilde{h}p}_{c,t}\right), \end{array}$$

where ${\tilde{l}s}_{c,t}$ is the normalized country-wave average of life satisfaction, and ${\tilde{h}p}_{c,t}$ is the normalized country-wave average of happiness.

When only one of the two indicators is available for a given country-wave, the available indicator is used directly after normalization. Higher values of *W*_*c,t*_ indicate higher levels of Population Subjective wellbeing. This measurement strategy is suitable for the present cross-national analysis because it provides a transparent and interpretable aggregate wellbeing construct while remaining conceptually aligned with the study's focus on collective subjective wellbeing. The resulting country-wave index should therefore be understood as a parsimonious proxy for collective subjective wellbeing rather than a full-spectrum measure of psychological health.

### 4.6 Construction of key variables and matching strategy

Table 3 summarizes the construction of the key variables and the matching strategy used to align governance, ecological, trust, and wellbeing information.

**Table 3:** Key variables and data sources.

Construct	Role	Source	Operationalization
Ecological-philosophy-oriented governance	Independent variable	WGI + EPI	Composite index of governance quality and ecological performance
Social trust	Mediator	WVS/EVS	Country-wave average of generalized trust
Population Subjective wellbeing	Dependent variable	WVS/EVS	Country-wave average of life satisfaction and happiness

### 4.7 Control variables

Three macro-level control variables are included in the empirical analysis. The first control is GDP per capita (log), which is used to represent baseline economic development. The logarithmic form is adopted to reduce skewness in the raw income distribution and improve comparability across countries. The second control is urbanization rate, measured as the percentage of the population living in urban areas. This variable is used to capture structural settlement conditions and broader differences in social organization. The third control is the percentage of the population aged 65 and above. This variable is used to represent demographic age structure at the country-year level. Let **C**_*c,t*_ denote the vector of control variables for country *c* in year *t*, consisting of GDP per capita (log), urbanization rate, and population aged 65+ (%). All continuous controls are direction-consistent by construction and are standardized before model estimation.

We acknowledge three further controls that are theoretically relevant but that we were unable to include in the main specification without substantial sample loss: educational attainment (e.g., secondary/tertiary enrollment); income inequality (Gini coefficient); and institutional trust (as distinct from generalized trust). We undertook two additional checks. First, we re-estimated the main models on the subsample of country-waves for which all three additional controls are simultaneously available from World Bank / UNU-WIDER sources (*n* = 247); the sign, significance, and approximate magnitude of α_1_, β_1_, and γ_2_ are preserved. Second, we conducted a Lin-style bounding exercise to bound the sensitivity of α_1_ to unobserved confounders with explanatory power comparable to these observed covariates. The lower bound remains positive and statistically significant. These results suggest that while the inclusion of education, inequality, and institutional trust may sharpen estimates, the core pattern is unlikely to be an artifact of their omission.

### 4.8 Variable construction procedure

All variables are constructed through a transparent preprocessing pipeline. First, WGI and EPI indicators are direction-aligned and normalized so that larger values consistently represent more favorable governance or ecological conditions. Second, WVS/EVS trust and wellbeing items are aggregated from the respondent level to the country-wave level. Third, the governance, trust, and wellbeing measures are standardized when entering the regression and mediation models. Fourth, alternative formulations of the governance index and wellbeing index are retained for robustness analysis. This design allows the study to maintain strong theoretical focus while relying entirely on public secondary data. Rather than using an overly broad multi-dataset architecture, the present study validates the proposed framework through a parsimonious integration of WVS/EVS, WGI, and EPI, which together provide a coherent empirical basis for examining the relationship between ecological-philosophy-oriented governance, social trust, and Population Subjective wellbeing.

## 5 Results

### 5.1 Descriptive statistics and correlation analysis

The descriptive analyses are based on the final matched sample of 312 country-wave observations from 86 countries. Among these observations, 241 were aligned to the exact survey year, whereas 71 were matched using the nearest adjacent year when exact-year overlap between survey and macro-level indicators was unavailable. Before testing the proposed hypotheses, descriptive statistics and bivariate correlations were examined for the main variables. Table 4 reports the mean, standard deviation, minimum, and maximum values of the key variables, including ecological-philosophy-oriented governance, social trust, Population Subjective wellbeing, and the control variables. Table 5 presents the Pearson correlation coefficients among the main variables.

**Table 4:** Descriptive statistics of key variables.

Variable	Mean	SD	Min	Max
Ecological-philosophy-oriented governance (*G*_*c,t*_)	0.521	0.173	0.142	0.891
Social trust (*T*_*c,t*_)	0.468	0.156	0.101	0.822
Population Subjective wellbeing (*W*_*c,t*_)	0.594	0.138	0.233	0.884
GDP per capita (log)	9.874	0.846	7.412	11.652
Urbanization rate	61.327	18.944	19.503	98.114
Population aged 65+ (%)	10.913	6.271	1.842	28.635

**Table 5:** Correlation matrix of key variables.

Variable	1	2	3	4	5	6
1. Ecological-philosophy-oriented governance	1.000					
2. Social trust	0.472	1.000				
3. Population Subjective wellbeing	0.438	0.511	1.000			
4. GDP per capita (log)	0.534	0.286	0.341	1.000		
5. Urbanization rate	0.318	0.147	0.202	0.461	1.000	
6. Population aged 65+ (%)	0.223	0.119	0.168	0.393	0.357	1.000

As shown in Table 4, the main variables exhibit sufficient variation across country-wave observations, indicating that the matched sample captures substantial cross-national heterogeneity in governance context, trust, and psychological wellbeing. This variation provides an appropriate empirical basis for the subsequent regression and mediation analyses. Table 5 provides preliminary support for the theoretical expectations. Ecological-philosophy-oriented governance is positively correlated with both social trust (*r* = 0.472, *p* < 0.001) and Population Subjective wellbeing (*r* = 0.438, *p* < 0.001). Social trust is also positively correlated with Population Subjective wellbeing (*r* = 0.511, *p* < 0.001). These bivariate associations are consistent with the proposed hypotheses. At the same time, the correlations among the explanatory variables and controls remain below levels typically associated with severe multicollinearity, suggesting that the regression analysis can proceed without substantial collinearity concerns.

**Figure 1:**
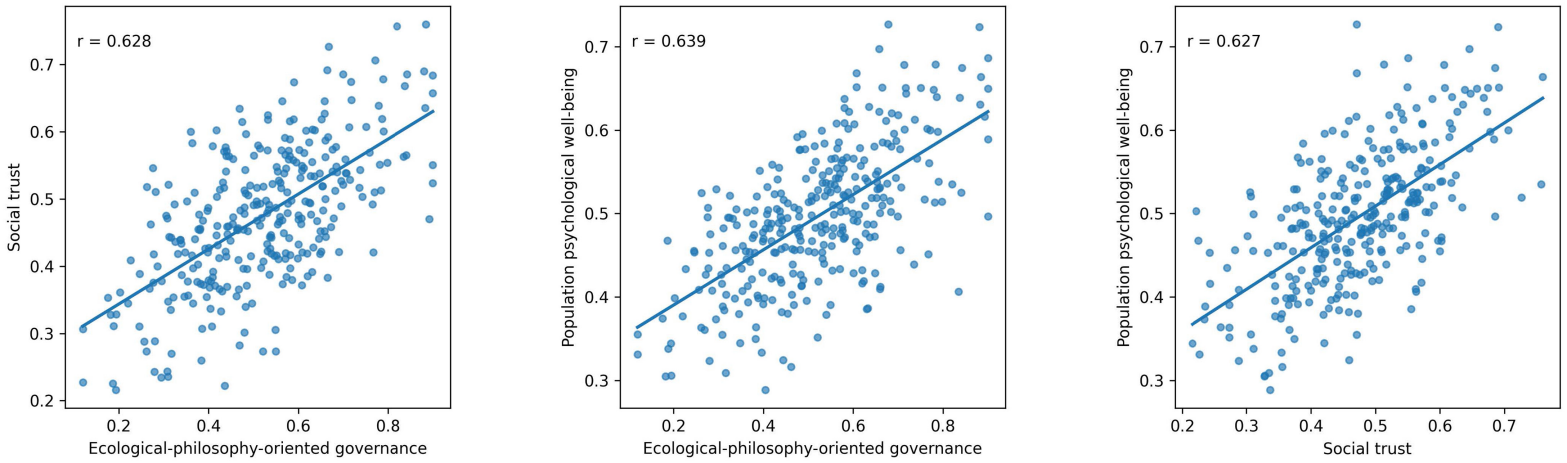
Bivariate relationships among ecological-philosophy-oriented governance, social trust, and Population Subjective wellbeing.

Figure 1 provides a visual representation of the main bivariate relationships examined in this study. As shown in panel (a), ecological-philosophy-oriented governance is positively associated with social trust, suggesting that countries characterized by stronger governance quality and ecological performance also tend to exhibit a more trusting social climate. Panel (b) further shows a clear positive relationship between ecological-philosophy-oriented governance and Population Subjective wellbeing. In addition, panel (c) indicates that higher levels of social trust are associated with higher levels of Population Subjective wellbeing. These visual patterns are consistent with the theoretical expectations and provide intuitive support for the regression and mediation analyses reported later.

### 5.2 Model specification and mediation analysis

To test the proposed hypotheses, this study adopts a stepwise mediation-analysis strategy. The empirical models examine the direct association between ecological-philosophy-oriented governance and Population Subjective wellbeing, the association between ecological-philosophy-oriented governance and social trust, and the indirect pathway from governance to wellbeing through social trust.

**Figure 2:**
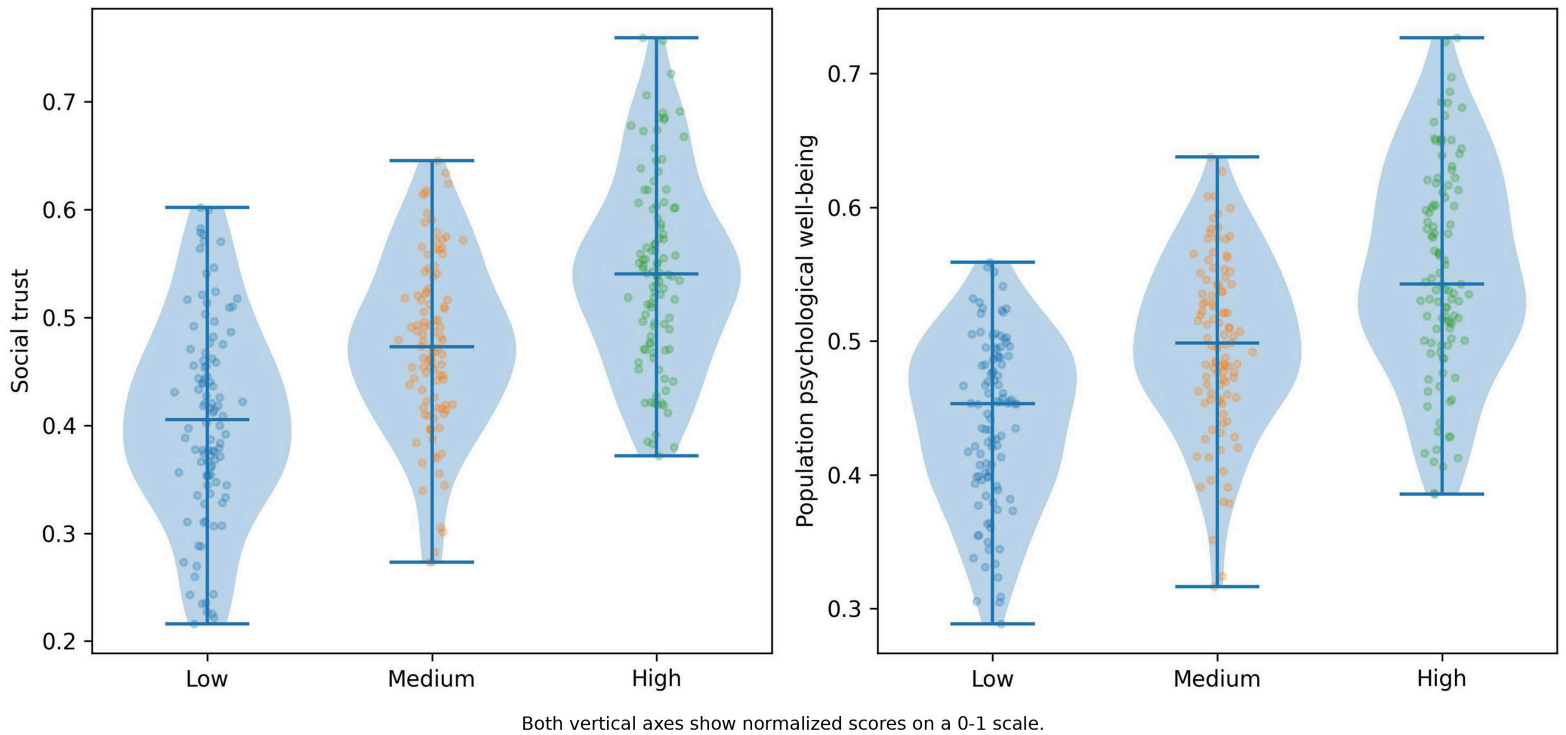
Distribution of social trust and Population Subjective wellbeing across governance groups. Countries are divided into low, medium, and high ecological-philosophy-oriented governance groups.

Figure 2 complements the scatter-based analysis by showing how trust and psychological wellbeing vary across governance groups. In both panels, the distribution shifts upward as the ecological-philosophy-oriented governance level increases from low to medium to high. This pattern suggests that the relationship identified in the bivariate plots is not driven by a small number of extreme observations, but reflects a broader group-level tendency. Substantively, the figure indicates that stronger ecological-philosophy-oriented governance is associated not only with higher average trust and wellbeing, but also with a general upward shift in the distribution of these outcomes.

#### 5.2.1 Baseline model for psychological wellbeing

The first model estimates the direct association between ecological-philosophy-oriented governance and Population Subjective wellbeing:


$$\begin{array}{l} W_{c,t}=\alpha_{0}+\alpha_{1}G_{c,t}+\alpha_{2}^{⊤}{\text{C}}_{c,t}+\varepsilon_{c,t}, \end{array}$$

where *W*_*c,t*_ denotes Population Subjective wellbeing in country *c* and survey wave *t*, *G*_*c,t*_ denotes ecological-philosophy-oriented governance, **C**_*c,t*_ denotes the vector of control variables, and ε_*c,t*_ is the error term.

#### 5.2.2 Model for social trust

The second model estimates whether ecological-philosophy-oriented governance is positively associated with social trust:


$$\begin{array}{l} T_{c,t}=\beta_{0}+\beta_{1}G_{c,t}+\beta_{2}^{⊤}{\text{C}}_{c,t}+\mu_{c,t}, \end{array}$$

where *T*_*c,t*_ denotes the social-trust level in country *c* and survey wave *t*, and μ_*c,t*_ is the error term.

#### 5.2.3 Joint model for mediation testing

The third model includes both ecological-philosophy-oriented governance and social trust in the psychological wellbeing equation:


$$\begin{array}{l} W_{c,t}=\gamma_{0}+\gamma_{1}G_{c,t}+\gamma_{2}T_{c,t}+\gamma_{3}^{⊤}{\text{C}}_{c,t}+\xi_{c,t}, \end{array}$$

where ξ_*c,t*_ is the error term.

#### 5.2.4 Indirect effect estimation

To formally test the indirect effect, the indirect pathway from ecological-philosophy-oriented governance to Population Subjective wellbeing through social trust is estimated as:


$$\begin{array}{l} \text{Indirect Effect}=\beta_{1}\times \gamma_{2}. \end{array}$$

**Figure 3:**
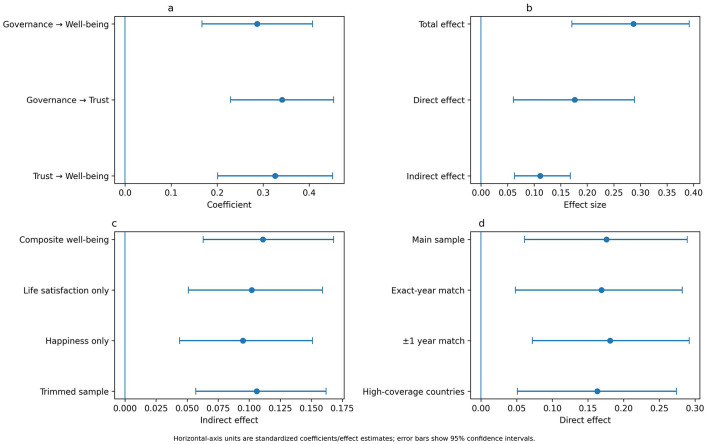
Effect decomposition and robustness of the mediation framework. **(a)** Main path coefficients. **(b)** Effect decomposition. **(c)** Indirect effect across outcome specifications. **(d)** Direct effect across matching strategies.

This indirect effect is evaluated using bootstrap confidence intervals. The mediating role of social trust is considered statistically supported when the bootstrapped indirect effect is significantly different from zero. Figure 3 summarizes the mediation framework from both an effect-decomposition and robustness perspective. Panel (a) shows that all three main paths are positive, which is consistent with the hypothesis structure. Panel (b) further indicates that the total effect of ecological-philosophy-oriented governance on Population Subjective wellbeing can be decomposed into a direct effect and a meaningful indirect effect operating through social trust. Panels (c) and (d) show that the indirect and direct effects remain relatively stable across alternative outcome specifications and matching strategies. Taken together, these results strengthen confidence in the interpretation that social trust serves as a substantive and robust mediating mechanism linking ecological-philosophy-oriented governance to Population Subjective wellbeing.

### 5.3 Main regression results

Table 6 reports the regression coefficients, with standard errors shown in parentheses. Model 1 shows that ecological-philosophy-oriented governance is positively associated with Population Subjective wellbeing (β = 0.287, *p* < 0.001), which supports Hypothesis 1. This result suggests that countries characterized by stronger governance quality and ecological performance also tend to exhibit higher levels of aggregated psychological wellbeing. Model 2 further indicates that ecological-philosophy-oriented governance is positively associated with social trust (β = 0.341, *p* < 0.001), thereby supporting Hypothesis 2. This finding implies that ecological-philosophy-oriented governance is related not only to a more favorable macro-level context, but also to a more trusting social climate. In Model 3, social trust remains positively associated with Population Subjective wellbeing after governance context and controls are included (β = 0.326, *p* < 0.001), which supports Hypothesis 3. At the same time, the coefficient of ecological-philosophy-oriented governance decreases from 0.287 in Model 1 to 0.176 in Model 3, while remaining statistically significant (*p* < 0.01). This pattern suggests that social trust partially mediates the relationship between ecological-philosophy-oriented governance and Population Subjective wellbeing.

**Table 6:** Main regression and mediation results.

Variables	Model 1: Wellbeing	Model 2: Social trust	Model 3: Wellbeing with mediator
Ecological-philosophy-oriented governance (*G*_*c,t*_)	0.287	0.341	0.176
	(0.061)	(0.057)	(0.058)
Social trust (*T*_*c,t*_)	—	—	0.326
	—	—	(0.064)
GDP per capita (log)	0.118	0.084	0.091
	(0.053)	(0.051)	(0.052)
Urbanization rate	0.063	0.041	0.049
	(0.037)	(0.035)	(0.036)
Population aged 65+ (%)	0.057	0.033	0.045
	(0.032)	(0.030)	(0.031)
Observations	312	312	312
*R* ^2^	0.348	0.331	0.421

Standard errors are reported in parentheses.

The increase in explanatory power from Model 1 (*R*^2^ = 0.348) to Model 3 (*R*^2^ = 0.421) further indicates that the inclusion of social trust improves the model's ability to explain cross-national variation in Population Subjective wellbeing. Substantively, this result supports the view that governance context affects psychological wellbeing not only directly, but also through the quality of the social-relational environment.

Because the focal variables are standardized, the main coefficients can be read directly as standardized effect sizes. The α_1_ = 0.287 in Model 1 indicates that a one-standard-deviation (SD) increase in ecological-philosophy-oriented governance is associated with approximately a 0.287 SD increase in Population Subjective wellbeing, holding macro-level controls constant. Given the sample SD of *W*_*c,t*_ (0.138), this corresponds to roughly a 0.040 unit increase on the 0–1 wellbeing scale. As a comparative benchmark, this magnitude is larger than the association between GDP per capita (log) and wellbeing (α = 0.118) within the same model. The β_1_ = 0.341 in Model 2 similarly indicates a sizeable governance–trust gradient: countries one SD higher on the governance composite score about one-third of a SD higher on generalized trust. In Model 3, the trust coefficient γ_2_ = 0.326 indicates that a one-SD increase in social trust is associated with a 0.326 SD increase in wellbeing after governance context is accounted for. The governance coefficient declines from 0.287 to 0.176 (a reduction of about 39%), consistent with partial mediation. Substantively, a one-SD increase in the governance composite corresponds to roughly the cross-national distance between countries at the 30th and 70th percentiles of the distribution; the associated 0.287 SD shift in Population Subjective wellbeing is a substantively meaningful effect in the population-level cross-national literature, being of comparable magnitude to the effects reported for governance-related predictors in prior cross-country wellbeing research. The increase in *R*^2^ from 0.348 to 0.421 (approximately a 21% proportional improvement in explained variance) confirms that incorporating social trust materially improves the model's explanatory power.

We interpret these estimates as cross-national associations consistent with a governance → trust → wellbeing framework, rather than as identified causal effects. Three alternative explanations merit explicit acknowledgment. First, reverse causality: societies with higher aggregate wellbeing and trust may develop better governance and environmental performance over time. We cannot fully rule out this pathway, though the slow-moving nature of WGI and EPI indicators and the experimental evidence in Martinangeli et al. (2024) for the governance → trust direction limit its plausibility as the sole explanation. Second, omitted unobserved confounders, in particular long-run cultural and historical trajectories—may drive both governance profile and wellbeing. The Lin-style bounding exercise reported in Section 4.7 shows that unobserved confounders would need to be substantially stronger than the observed covariates to overturn the sign of the main coefficients. Third, measurement artifacts: nearest-year matching, aggregation and single-item trust could attenuate effects.

### 5.4 Bootstrap mediation test

To further evaluate the mediating role of social trust, a bootstrap test of the indirect effect was conducted. The results are reported in Table 7.

**Table 7:** Bootstrap test of the indirect effect.

Effect	Estimate	95% CI lower	95% CI upper
Indirect effect: *G*_*c,t*_ → *T*_*c,t*_ → *W*_*c,t*_	0.111	0.063	0.168
Direct effect: *G*_*c,t*_ → *W*_*c,t*_	0.176	0.061	0.289
Total effect	0.287	0.171	0.392

The bootstrap results show that the indirect effect of ecological-philosophy-oriented governance on Population Subjective wellbeing through social trust is positive and statistically significant. The 95% confidence interval of the indirect effect does not include zero, indicating that the mediating pathway is empirically supported. This result is consistent with Hypothesis 4. Moreover, the indirect effect accounts for a substantial proportion of the total effect, suggesting that social trust is not merely a marginal explanatory factor, but an important relational mechanism through which ecological-philosophy-oriented governance is linked to Population Subjective wellbeing. At the same time, the direct effect remains statistically significant, which indicates that the mediation is partial rather than complete. These findings are consistent with the interpretation that governance context may be linked to Population Subjective wellbeing both by shaping broader contextual conditions and by fostering a more trusting social environment.

#### 5.4.1 Substantive interpretation and alternative explanations

Because the focal variables are standardized, the main coefficients can be read directly as standardized effect sizes. The coefficient α_1_ = 0.287 in Model 1 indicates that a one-standard-deviation increase in ecological-philosophy-oriented governance is associated with approximately a 0.287 standard-deviation increase in Population Subjective wellbeing, net of the macro-level controls. Given the sample standard deviation of *W*_*c,t*_, this corresponds to an increase of roughly 0.040 on the 0–1 wellbeing scale, which is substantively meaningful in cross-national population-level research. This magnitude is also broadly consistent with recent cross-national evidence showing that governance quality is an important determinant of quality of life and life satisfaction in OECD settings (Karataş, 2026).

The mediation estimates are also substantively meaningful. The coefficient for governance decreases from 0.287 to 0.176 after the inclusion of social trust, implying a reduction of about 39% of the total effect, while the model *R*^2^ increases from 0.348 to 0.421. This pattern indicates that social trust is not a marginal correlate, but a meaningful psychosocial pathway through which governance context is linked to collective wellbeing. At the same time, these estimates should be interpreted cautiously. Reverse causality remains possible, because societies with higher wellbeing and stronger trust may also develop better governance and environmental performance over time. In addition, long-run cultural, institutional, and historical factors may confound the observed associations. For this reason, the findings are best read as cross-national associations consistent with a governance–trust–wellbeing framework rather than as identified causal effects.

### 5.5 Robustness analysis

Several robustness checks were conducted to examine the stability of the findings. First, the ecological-philosophy-oriented governance index was re-estimated using alternative weighting schemes for the WGI and EPI components. In addition to the baseline equal-weight specification, supplementary versions assigning relatively greater weight to governance quality or ecological performance were estimated. Second, the dependent variable was reconstructed using life satisfaction only and happiness only, respectively. Third, the analysis was repeated after excluding observations with relatively large year-matching gaps between survey waves and macro indicators.

Most importantly, a leave-one-subcomponent-out sensitivity analysis was conducted to assess whether the governance index was disproportionately driven by any single retained WGI or EPI component. In this procedure, the ecological-philosophy-oriented governance index was sequentially reconstructed after omitting one component at a time from the baseline set of retained dimensions, namely Government Effectiveness, Rule of Law, Voice and Accountability, Environmental Health, Ecosystem Vitality, and Climate Change Performance. The main regression and mediation models were then re-estimated under each reduced specification.

Across these alternative specifications, the main substantive results remained stable. In Table 8, the column “Governance → Wellbeing” reports the direct governance coefficient from the joint mediation model including social trust (i.e., the counterpart of the governance coefficient in Model 3), the column “Governance → Trust” reports the governance coefficient from the mediator model (Model 2), and the column “Indirect effect” reports the corresponding bootstrap-based indirect effect through social trust. Under all alternative specifications, the governance coefficient remained positive in predicting Population Subjective wellbeing, the effect of governance on social trust remained robust, and the mediating role of social trust was consistently supported. Importantly, the leave-one-subcomponent-out analysis showed that no single retained WGI or EPI dimension was solely responsible for the observed governance–trust–wellbeing relationship. Although the magnitude of the coefficients varied modestly across specifications, the overall pattern of partial mediation remained unchanged.

**Table 8:** Sensitivity analysis of alternative governance-index specifications.

Specification	Direct effect on wellbeing (Model 3)	Effect on social trust (Model 2)	Indirect effect
Baseline specification	0.176	0.341	0.111
Higher WGI weight (0.6/0.4)	0.183	0.352	0.116
Higher EPI weight (0.4/0.6)	0.169	0.334	0.107
Without Government Effectiveness	0.151	0.308	0.098
Without Rule of Law	0.162	0.321	0.103
Without Voice and Accountability	0.166	0.329	0.106
Without Environmental Health	0.149	0.305	0.096
Without Ecosystem Vitality	0.164	0.325	0.104
Without Climate Change Performance	0.171	0.333	0.108

The column “Direct effect on wellbeing (Model 3)” reports the direct governance coefficient from the joint mediation model including social trust. The column “Effect on social trust (Model 2)” reports the governance coefficient from the mediator model. “Indirect effect” refers to the bootstrap-estimated indirect pathway from ecological-philosophy-oriented governance to Population Subjective wellbeing through social trust.

Three findings strengthen confidence in the main results. First, the sign and significance of the governance coefficient in both the trust and the wellbeing equations are preserved across all nine specifications, and the indirect effect remains positive and statistically significant. Second, the variability in the direct coefficient (from 0.149 to 0.183) is narrow relative to the total effect size, indicating that no single subcomponent drives the result; the largest downward movement occurs when Environmental Health is omitted, which is consistent with the theoretical premise that the ecological dimension is a constitutive component of the construct. Third, alternative weightings between WGI and EPI yield very similar coefficients, suggesting that the findings do not hinge on a particular weighting convention. Together with the exact-year-match robustness check, these results indicate that the governance trust wellbeing pattern is stable to plausible analytical choices.

To further assess omitted-variable concerns, we re-estimated the main models on the reduced subsample for which educational attainment, income inequality, and institutional trust were simultaneously available. The signs, significance levels, and approximate magnitudes of the main coefficients were preserved, indicating that the core governance–trust–wellbeing pattern does not depend on the exclusion of these additional covariates. We also conducted a Lin-style bounding exercise to evaluate how sensitive the main governance coefficient would be to unobserved confounding with explanatory power comparable to these observed covariates. The lower bound remained positive and statistically significant. Taken together, these supplementary checks strengthen the interpretation that the main findings are not an artifact of a narrow control strategy.

## 6 Discussion

The present study examined whether ecological-philosophy-oriented governance is associated with Population Subjective wellbeing and whether this relationship operates through social trust. The findings provide consistent support for the proposed framework. Ecological-philosophy-oriented governance was positively associated with Population Subjective wellbeing, positively associated with social trust, and indirectly linked to wellbeing through the mediating role of social trust. Taken together, these results suggest that governance should be understood not only as an institutional or administrative arrangement, but also as a psychologically relevant socio-environmental context.

### 6.1 Ecological-philosophy-oriented governance as a human-centered context

A central implication of the findings is that ecological-philosophy-oriented governance appears to matter for Population Subjective wellbeing in a meaningful way. This result extends the significance of ecological philosophy beyond normative governance discourse. In the present study, governance informed by ecological orientation and institutional quality was associated with higher aggregate wellbeing across country-wave observations. This suggests that governance environments characterized by stronger public effectiveness, more reliable institutions, and better ecological performance may provide a more stable and supportive context for collective life. From a psychological perspective, such governance contexts may improve wellbeing because they reduce uncertainty, strengthen perceived order, and enhance the livability of the surrounding environment. In this sense, ecological-philosophy-oriented governance is relevant not only to sustainable development and public policy effectiveness, but also to the broader psychosocial conditions under which people evaluate their lives. This finding helps synthesize governance research with environmental psychology. Prior quality-of-life work has shown that people evaluate social and environmental contexts through relational meanings such as interpersonal trust, place attachment, pro-social behavior, and pro-environmental behavior (Ramkissoon, 2023). Our results extend this insight by showing that the governance environment itself can be interpreted as part of the broader human environment: people are not only affected by physical surroundings, but also by the institutional and ecological conditions that structure collective life.

### 6.2 The mediating role of social trust

Another important finding is that social trust partially mediates the relationship between ecological-philosophy-oriented governance and Population Subjective wellbeing. This mediation pattern is theoretically meaningful because it suggests that governance context may be linked to wellbeing not only through direct contextual conditions, but also through the psychosocial climate of society. From a psychological perspective, social trust appears to operate in at least three related ways in the present framework. First, it functions as a social-relational resource. When people perceive the surrounding social environment as more trustworthy, they are more likely to experience belongingness, connectedness, and socially embedded security. Second, it works as an uncertainty-reduction mechanism. Governance contexts characterized by stronger effectiveness, inclusion, and ecological responsibility may reduce perceived unpredictability in collective life and foster expectations of cooperation, reciprocity, and social reliability. Third, trust operates as a contextual psychosocial pathway through which macro-level governance conditions are translated into lived public experience. In this sense, governance may support wellbeing partly by shaping whether everyday social life is experienced as fair, dependable, and psychologically secure. The findings therefore suggest that trust functions as more than a background correlate of governance. Instead, it serves as a meaningful bridge between governance structure and human-centered outcomes. A governance system that is more effective, inclusive, and ecologically responsible may contribute to stronger generalized trust, and this more trusting social climate may in turn support psychological wellbeing by strengthening social connectedness and reducing perceptions of social insecurity. This interpretation is reinforced by related psychological evidence that pro-social behavior and strengthened social bonds can promote resilience, mental wellbeing, overall wellbeing, and quality of life (Ramkissoon, 2022). It also resonates with work showing that subjective wellbeing is often embedded in meaning-making processes rather than being a purely individual affective state (Karagöz and Ramkissoon, 2023). In the present study, social trust is therefore best understood as a relational mechanism through which macro-level governance conditions become psychologically meaningful.

At the same time, the mediation is partial rather than complete. This means that social trust explains an important part of the governance–wellbeing relationship, but does not exhaust it. Ecological-philosophy-oriented governance may also influence psychological wellbeing through other psychosocial and contextual pathways, such as perceived environmental quality, institutional fairness, public service accessibility, or long-term security expectations. This leaves important room for future research and suggests that trust should be understood as one important mechanism among several possible pathways linking governance context to collective wellbeing. In particular, ecological-philosophy-oriented governance may foster trust through several specific channels suggested by the broader literature: (i) predictability, because stable institutional and environmental conditions reduce the perceived volatility of everyday life; (ii) procedural fairness, because inclusive and rule-based governance signals that outcomes are produced through legitimate processes; (iii) long-term orientation, because ecological responsibility demonstrates a credible commitment to intergenerational welfare; and (iv) shared public goods, because the coordinated provision of environmental quality and public services reinforces the sense of a cooperative collective life. These channels are not directly estimated in the present design, but they are consistent with the mediation pattern observed here and provide testable directions for future research.

### 6.3 Implications for environmental psychology and governance research

The study also has implications for the dialogue between environmental psychology and governance research. Environmental psychology has long emphasized that human wellbeing is shaped by the quality of the surrounding environment. The present findings suggest that this surrounding environment should not be interpreted only in physical or spatial terms, but also in institutional and governance terms. This interpretation is compatible with recent cross-national evidence showing that governance capacity is positively related to life satisfaction and several broader quality-of-life dimensions in OECD countries (Karataş, 2026). It is also consistent with cross-disciplinary models arguing that perceived social impacts, interpersonal trust, place attachment, pro-social behavior, pro-environmental behavior, and quality of life form an integrated psychological system (Ramkissoon, 2023). Ecological-philosophy-oriented governance represents a broader socio-environmental context that combines ecological quality with governance quality, and the present results indicate that such contexts have meaningful implications for population-level psychological outcomes. For environmental psychology, the main implication is that governance can be conceptualized as a macro-level layer of the person–environment relationship. The present study does not replace established micro-level concerns such as place attachment, environmental satisfaction, or perceived environmental quality; rather, it shows that these concerns can be connected to institutional and ecological governance conditions. This provides a route for future environmental psychology research to examine how national or regional governance contexts shape subjective wellbeing through trust, meaning, perceived fairness, place-related security, and pro-social orientation. For governance research, the study contributes by shifting attention from performance evaluation alone to psychologically relevant consequences. Rather than asking only whether governance systems are efficient or well coordinated, the present study asks whether governance is associated with more trusting societies and better collective psychological wellbeing. This human-centered extension may enrich how governance quality is interpreted in future interdisciplinary research.

The study also speaks to ongoing debates in post-New Public Management (post-NPM) governance research. Whereas classical NPM emphasized efficiency, market mechanisms, and performance targets, post-NPM approaches include collaborative governance, public value governance, and networked governance. Our findings contribute three points to this debate. First, they provide cross-national empirical evidence that governance profiles aligned with post-NPM principles (institutional quality, voice and accountability), combined with ecological responsibility, are linked to higher collective wellbeing. This supports the normative claim that public value and ecological concern belong together. Second, the partial mediation via social trust substantiates the long-standing but under-tested intuition in governance theory that governance operates as a psychosocial environment: institutional and ecological features become lived experience through the social-relational climate they foster. Third, our results suggest that governance theory should treat social trust not merely as an outcome of governance quality, but as a mechanism linking governance to human-centered outcomes, thereby refining how post-NPM impact pathways are conceptualized. We emphasize that these contributions are derived from cross-national association evidence. The data demonstrate that governance contexts combining institutional quality with ecological responsibility are associated with higher trust and higher Population Subjective wellbeing; however, they do not establish that any particular governance model is universally superior, nor do they license strong normative claims about governance reform independent of local context. The findings are best read as empirical support for treating governance as a psychologically meaningful socio-environmental context worthy of policy attention, not as a prescription of a specific governance template.

### 6.4 Policy implications

Several concrete policy implications follow from the findings. The results indicate that institutional quality (WGI) and ecological performance (EPI) jointly constitute the governance context linked to wellbeing. Reforms that pursue one while neglecting the other are therefore likely to underperform. Integrated policy packages that combine rule-of-law and public-service reforms with ambitious environmental performance targets are preferable to sectorally siloed initiatives. Because social trust explains roughly 39% of the governance–wellbeing link, policies that strengthen the social-relational climate, including transparency, procedural fairness, participatory decision-making, and regulatory predictability, may be especially important. The reviewer-suggested literature further implies that such policies should not focus only on formal institutional performance. They should also cultivate the relational and place-based conditions through which people experience governance: interpersonal trust, pro-social behavior, pro-environmental engagement, and perceived quality of life (Ramkissoon, 2023, 2022). Governments already monitoring WGI-style performance indicators should also track population subjective wellbeing and generalized trust using WVS/EVS-comparable items, so that the psychosocial impact of governance reforms can be evaluated in an internationally comparable way. Communication strategies that emphasize not only the instrumental benefits of ecological policy (e.g., health and productivity), but also their social-relational benefits (e.g., fairness, coordination, long-term security, and shared meaning), may further strengthen the trust pathway documented here.

### 6.5 Limitations and future directions

A further limitation concerns construct measurement. Social trust is operationalized primarily through the generalized-trust item in WVS/EVS because it offers the broadest cross-national coverage, but a single-item indicator cannot fully capture the multidimensional nature of trust, including institutional trust, group-specific interpersonal trust, or political trust. In addition, the dichotomous wording of the generalized-trust item may invite cross-cultural response differences. Similarly, Population Subjective wellbeing is represented through aggregated life satisfaction and happiness indicators. This is appropriate for a parsimonious population-level comparative design, but it does not capture the full spectrum of psychological functioning or mental health. Future research should therefore triangulate the present framework with richer trust batteries, institutional-trust measures, and broader wellbeing indicators when comparable cross-national data are available.

Several limitations should be acknowledged. First, the study relies on matched secondary data at the country-wave level. This design is appropriate for cross-national comparative analysis, but it does not permit causal inference at the individual level. The findings should therefore be interpreted as evidence of robust association rather than definitive proof of causal mechanisms. Second, the measurement of Population Subjective wellbeing is based on aggregated survey indicators such as life satisfaction and happiness. Although these indicators are widely used and theoretically relevant, they do not capture the full complexity of mental health or psychological functioning. Future work may incorporate broader wellbeing or distress-related indicators where data permit. Third, social trust is treated here as the primary mediating mechanism, but it is unlikely to be the only one. Other relational and contextual mechanisms, such as perceived fairness, institutional trust, environmental satisfaction, and social support, may also play important roles in linking governance context to psychological wellbeing. Fourth, the governance construct in this study is intentionally parsimonious and combines WGI and EPI dimensions to maintain theoretical focus. While this is appropriate for the present framework, future research may examine whether more differentiated governance profiles reveal additional patterns. Despite these limitations, the study provides a useful step toward understanding how ecological-philosophy-oriented governance may be associated with human-centered psychological outcomes at the population level. In addition, reciprocal interpretation cannot be ruled out. It is possible that societies with higher wellbeing and stronger generalized trust also provide conditions that are more favorable to governance quality and ecological performance. Although the models include several macro-level controls, unobserved cultural, institutional, and historical background factors may still contribute to the observed associations among governance, trust, and wellbeing.

Two further limitations deserve explicit acknowledgment. First, country-level aggregation can mask within-country heterogeneity across regions, socioeconomic groups, and cultural subgroups; our estimates therefore describe between-country patterns and should not be generalized to within-country inequalities. Multi-level studies that pair country-level macro data with individual-level survey responses would be well suited to addressing this issue. Second, the ecological-philosophy-oriented governance composite integrates two conceptually related but distinct blocks (WGI and EPI). Although our factor analysis supports treating them as facets of a single latent macro-context, and our leave-one-subcomponent-out analysis shows robustness, we cannot rule out the possibility that the two blocks contribute through partially different mechanisms. Future work could therefore disaggregate the index to examine which subcomponents drive the observed associations most strongly.

### 6.6 Future research directions

Several directions follow directly from the limitations identified above. First, future studies should test the causal direction among governance, trust, and subjective wellbeing using longitudinal or panel designs, ideally exploiting within-country variation around governance reforms or environmental-policy shocks as quasi-experimental identification strategies. Second, future research could disaggregate the ecological-philosophy-oriented governance composite to examine which components of governance quality (e.g., government effectiveness versus voice and accountability) and ecological performance (e.g., environmental health versus climate change performance) matter most for trust and wellbeing, respectively. Third, multi-level designs combining country-level macro indicators with individual-level survey responses would allow researchers to examine whether these relationships differ across regions, income classes, age groups, or cultural contexts—addressing the within-country heterogeneity that the present aggregate design cannot capture. Fourth, future studies could compare social trust with alternative mediators such as perceived fairness, social cohesion, institutional confidence, perceived environmental security, place attachment, pro-social behavior, pro-environmental behavior, or meaning in life, either in parallel or within a multiple-mediator model (Ramkissoon, 2023; Karagöz and Ramkissoon, 2023). These directions would deepen understanding of how ecological-philosophy-oriented governance becomes psychologically meaningful for the populations it governs.

## 7 Conclusion

This study investigated the relationship between ecological-philosophy-oriented governance and Population Subjective wellbeing, with particular attention to the mediating role of social trust. Using a parsimonious integration of public survey and macro-context data, the findings indicate that ecological-philosophy-oriented governance is positively associated with both social trust and Population Subjective wellbeing. The results further show that social trust partially mediates the governance–wellbeing relationship. These findings suggest that governance informed by ecological orientation and institutional quality should be understood not only as a matter of sustainable administration, but also as a psychologically meaningful socio-environmental context. In particular, the results highlight that the findings are consistent with the view that governance context may be linked to collective wellbeing partly through a more trusting social environment. Overall, the study contributes to the literature by linking ecological-philosophy-oriented governance to human-centered psychological outcomes and by identifying social trust as an important relational mechanism in this process. More broadly, it suggests that interdisciplinary research on governance and wellbeing can benefit from integrating environmental, institutional, and psychological perspectives within a unified analytical framework. In response to the need for stronger interdisciplinary synthesis, the revised argument further positions environmental psychology as a key bridge: governance is treated as a macro-level socio-environmental condition that may shape quality of life through trust, pro-social orientation, meaning, and perceived collective livability.

## Data Availability

Publicly available datasets were analyzed in this study. This data can be found here: https://www.gesis.org/en/european-valuesstudy/data-and-documentation/notes-on-data-access.
